# Drought-responsive genes in tomato: meta-analysis of gene expression using machine learning

**DOI:** 10.1038/s41598-023-45942-2

**Published:** 2023-11-08

**Authors:** Rabiul Haq Chowdhury, Fatiha Sultana Eti, Rayhan Ahmed, Shipan Das Gupta, Pijush Kanti Jhan, Tofazzal Islam, Md. Atiqur Rahman Bhuiyan, Mehede Hassan Rubel, Abul Khayer

**Affiliations:** 1 Department of Agriculture, Noakhali Science and Technology University, Sonapur, 3814 Bangladesh; 2 Faculty of Agricultural Sciences, Georg-August Universität Göttingen, Büsgenweg 5, 37077 Göttingen, Germany; 3 Department of Biotechnology and Genetic Engineering, Noakhali Science and Technology University, Sonapur, 3814 Bangladesh; 4 Institute of Biotechnology and Genetic Engineering (IBGE), Bangabandhu Sheikh Mujibur Rahman Agricultural University, Gazipur, 1706 Bangladesh; 5 Present Address: Computomics GmbH, Eisenbahnstr. 1, 72072 Tübingen, Germany

**Keywords:** Computational models, Gene ontology, Machine learning

## Abstract

Plants have diverse molecular mechanisms to protect themselves from biotic and abiotic stressors and adapt to changing environments. To uncover the genetic potential of plants, it is crucial to understand how they adapt to adverse conditions by analyzing their genomic data. We analyzed RNA-Seq data from different tomato genotypes, tissue types, and drought durations. We used a time series scale to identify early and late drought-responsive gene modules and applied a machine learning method to identify the best responsive genes to drought. We demonstrated six candidate genes of tomato viz. Fasciclin-like arabinogalactan protein 2 (*FLA2*), Amino acid transporter family protein (*ASCT*), Arginine decarboxylase 1 (*ADC1*), Protein NRT1/PTR family 7.3 (*NPF7.3*), BAG family molecular chaperone regulator 5 (*BAG5*) and Dicer-like 2b (*DCL2b*) were responsive to drought. We constructed gene association networks to identify their potential interactors and found them drought-responsive. The identified candidate genes can help to explore the adaptation of tomato plants to drought. Furthermore, these candidate genes can have far-reaching implications for molecular breeding and genome editing in tomatoes, providing insights into the molecular mechanisms that underlie drought adaptation. This research underscores the importance of the genetic basis of plant adaptation, particularly in changing climates and growing populations.

## Introduction

The growing global population and the worsening effects of climate change present a formidable challenge to the field of agriculture. To meet the increasing demand for food, agricultural scientists must find ways to increase production despite the adverse effects of climate change. One of the major factors that can reduce crop yields is water scarcity, which is a common problem in many regions, such as South Asia, where prolonged summers lead to low water availability and moisture content in the soil^1,2^. The prolonged stress can severely diminish plant growth and productivity, and cause the accumulation of reactive oxygen species (ROS) in the plant. Lower carbon fixation due to stomata closure can lead to reduced NADP + regeneration through the Calvin cycle, which, when coupled with changes in chlorophyll production, can result in an increased production of ROS in water-stressed plants^3–6^. The accumulation of ROS can lead to a cascade of harmful effects, including peroxidation of lipids, protein oxidation, enzymatic activity inhibition, oxidative damage to nucleic acids, and ultimately, cell death^6,7^. These consequences can have serious implications for agriculture, as they can ultimately lead to decreased crop yields and food shortages. Therefore, finding effective ways to mitigate the impact of water scarcity on crops is crucial to ensure food security in the face of climate change.

Tomatoes are a widely cultivated crop throughout the world, originating in southern America as a cultivated solanaceous plant known as *Solanum lycopersicum*. Despite its origins, tomatoes are now produced and consumed worldwide, with demand slightly outstripping production^7–9^. Due to their relatively short life cycle, easy cultivation, and simple genetics with lower gene duplication, tomatoes are considered to be a standard model of vegetables^1,8,10^. Molecular studies of tomatoes can also provide valuable insights into other related species^8^. While tomatoes are economically important, there is still much to learn about their molecular responses to various abiotic stresses, such as water stress.

Next-generation sequencing technologies have rapidly become the preferred method for characterizing and quantifying entire genomes. One powerful application of these high-throughput sequencing methods is RNA sequencing (RNA-Seq), which allows researchers to gain insight into the transcriptome of cells. Compared to previous approaches such as Sanger sequencing and microarray-based methods, RNA-Seq provides higher resolution and greater sensitivity for characterizing the dynamic nature of the transcriptome. In addition to quantifying gene expression, RNA-Seq data can facilitate the discovery of novel transcripts, identification of alternative splicing events, and detection of allele-specific expression. Recent advances in the RNA-Seq workflow, including improvements in sample preparation, library construction, and data analysis, have enabled researchers to uncover even more of the functional complexity of transcription^11,12^.

The analysis of RNA-Seq data is a significant challenge due to its complex and high-dimensional nature. However, in recent years, machine learning has emerged as a successful approach in various biological contexts. Machine learning algorithms have demonstrated impressive efficiency in handling large datasets that possess characteristics such as noise, high dimensionality, and incompleteness. One notable advantage is that these algorithms require minimal assumptions about the underlying probability distributions and generation methods of the data. In contrast to traditional statistical approaches that focus on inference, machine learning methods prioritize prediction, providing greater flexibility to accommodate diverse data characteristics. Machine learning plays a crucial role in the analysis of biological data, particularly RNA-Seq data, due to its capability to handle nonlinearity. By integrating RNA-Seq data with machine learning techniques, nitrogen-responsive genes in both Arabidopsis and maize were successfully identified and functionally validated, conducting experiments in both field and greenhouse environments^13,14^. Linear models often fall short of capturing the intricate relationships present in biological datasets. Conversely, machine learning algorithms excel at capturing and utilizing the complex patterns inherent in such data. This ability enhances our understanding and interpretation of RNA-Seq data, enabling more accurate predictions and deeper insights into biological phenomena^15,16^.

In this research, we gathered transcriptomics data from various tomato genotypes subjected to different drought conditions. The experiment encompassed diverse genotypes and included six time points representing varying durations of drought, along with corresponding control groups. Our analysis focused on identifying genes that exhibited differential expression, allowing us to capture both early and late responsive genes to drought. To achieve this, we employed machine learning models, systematically exploring the dataset to uncover the genes most affected by drought in tomato plants. Using the identified genes, we constructed gene association networks and determined their interactors as drought-responsive. Additionally, we observed that the chromosomal positions of these genes overlapped with previously reported drought-responsive Quantitative Trait Loci (QTLs) in tomato.

**Figure 1:**
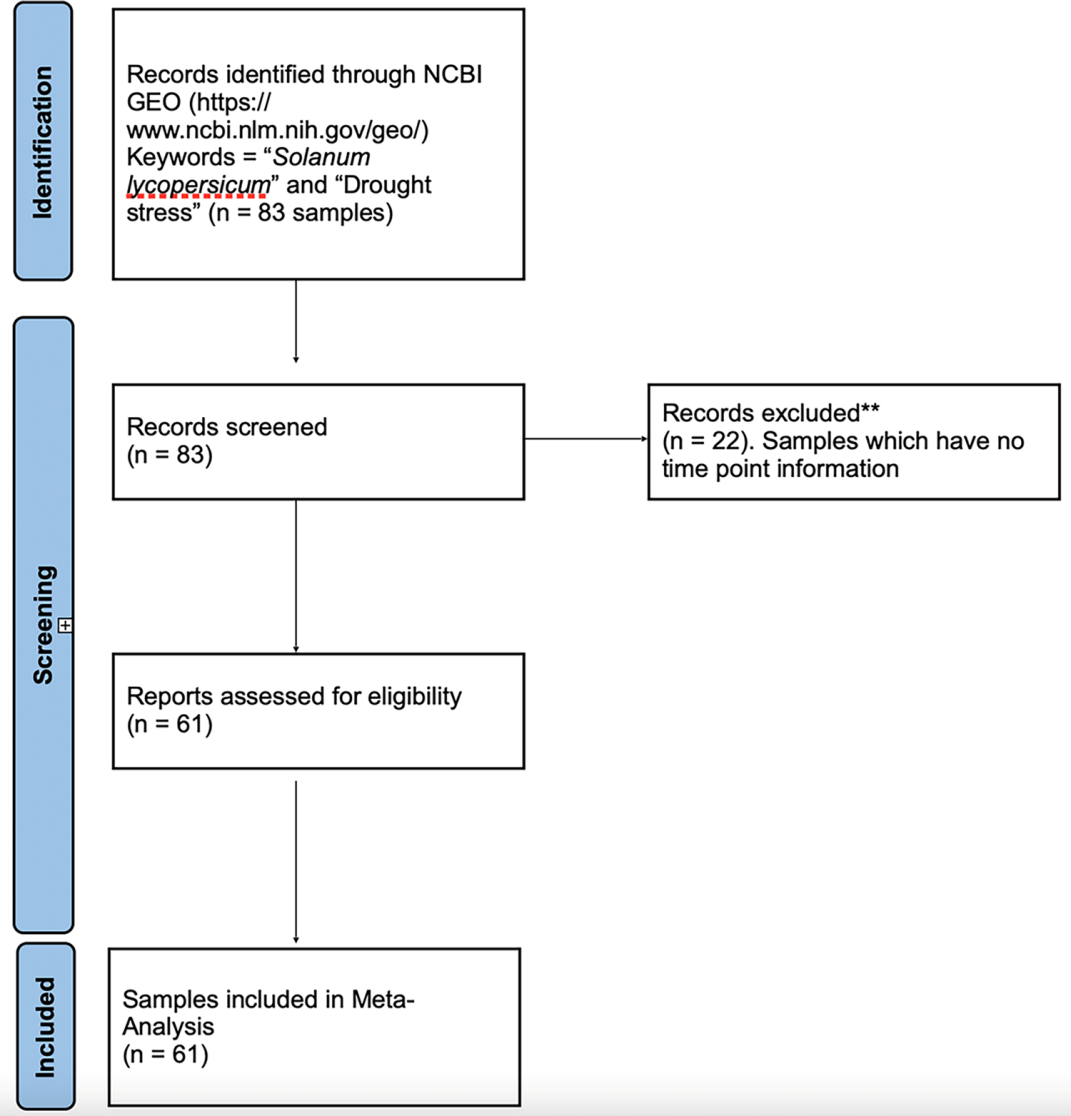
Flow chart showing the transcriptomics data collection. The samples mean the number of records are retrieved from the databases.

The study has also investigated differentially expressed genes and enriched Gene Ontology (GO) categories in tomatoes subjected to prolonged drought stress and subsequent rehydration. These studies revealed that down-regulated genes after drought stress were enriched in GO categories related to photosynthesis and cell proliferation, whereas upregulated genes belonged to GO categories more directly associated with stress responses^3,7,10^. However, a comprehensive examination of genetic responses in tomato plants to drought stress is still lacking. Therefore, the primary objective of our study was to employ machine learning techniques to investigate the responsive genes of tomato plants under prolonged water deficit conditions. Through our systematic approach, we aimed to identify the genes specifically responsive to this particular stress condition in plants (Fig. 1).

## Results

### Differentially expressed gene patterns

**Figure 2:**
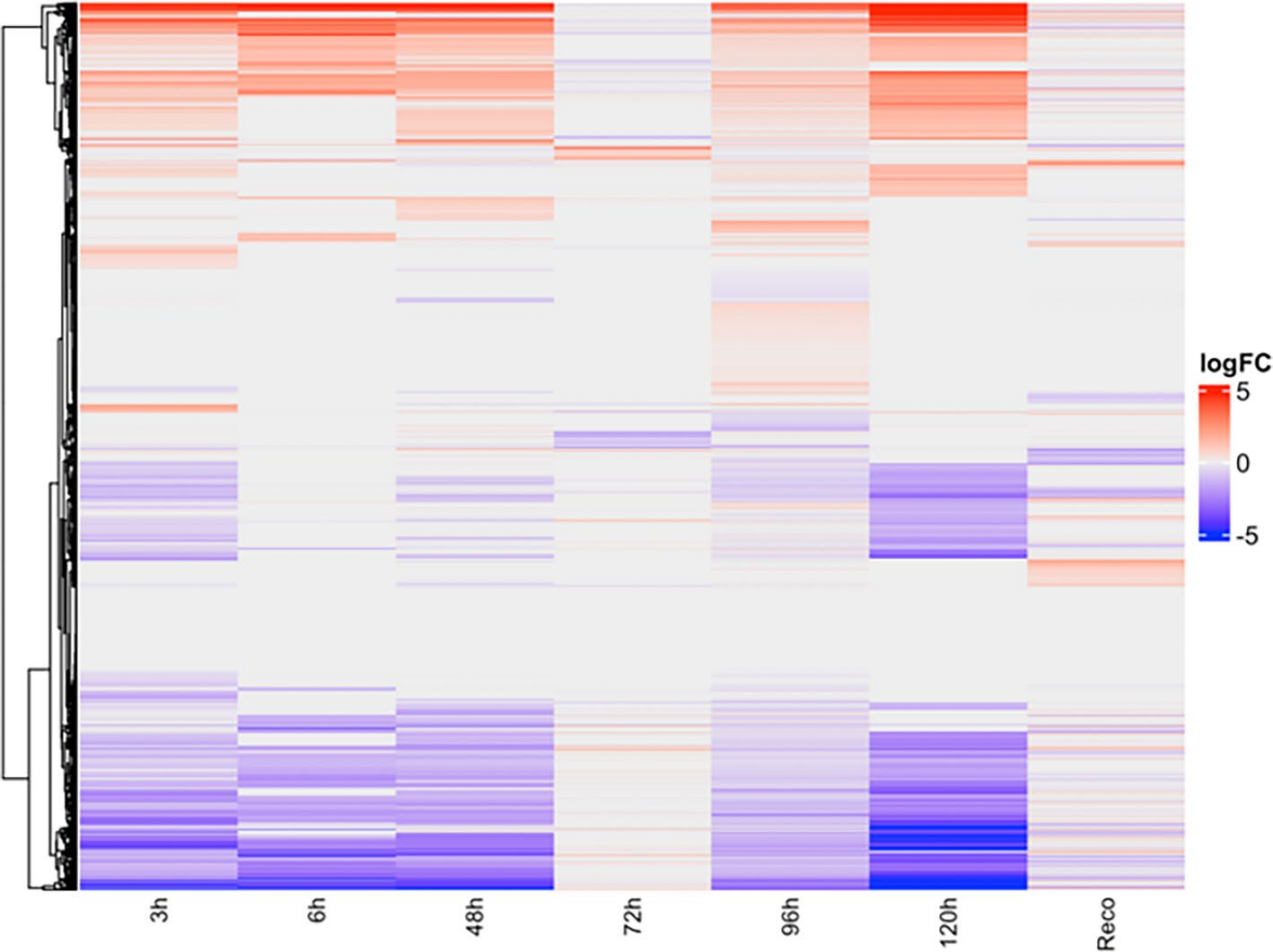
Heatmap showing differentially expressed genes under drought stress in different time series data. logFC was calculated by comparing respective controls. The red color in the heatmap denotes upregulation and the blue color denotes downregulation. The Y-axis denotes the differentially expressed genes.

The expression of genes can vary over time in response to different stimuli, and the identification of DEGs can provide insights into the underlying biological processes. In this study, the expression of genes in a time series scale was analyzed, and the results were visualized in Fig. 2.

**Figure 3:**
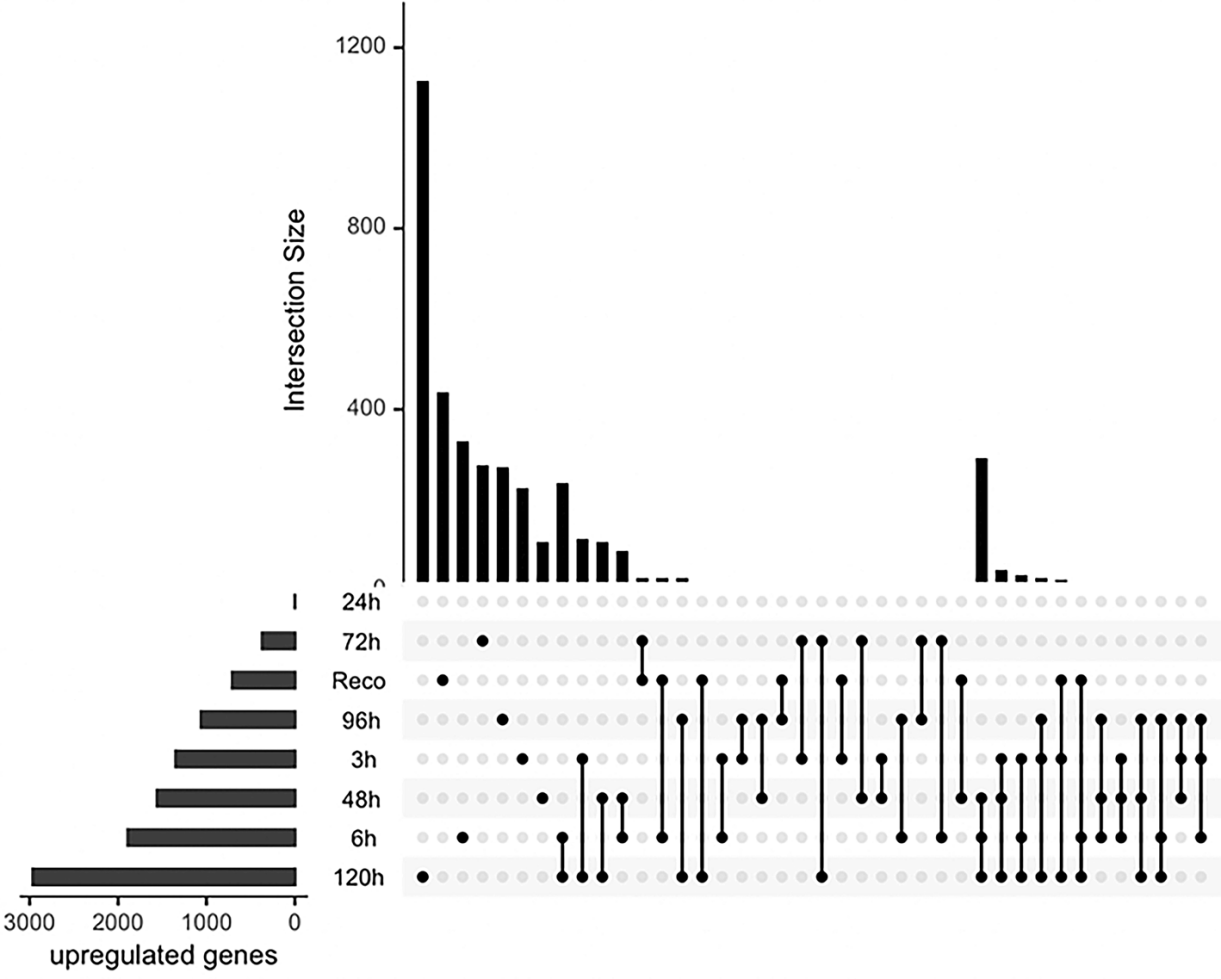
Upset plot showing differentially expressed upregulated gene numbers under drought stress in different time series data. Vertical bars show the unique upregulated genes per time point. Horizontal bars display the total number of upregulated genes per time point. Dots connecting time points denote the unique upregulated genes to respective time points.

**Figure 4:**
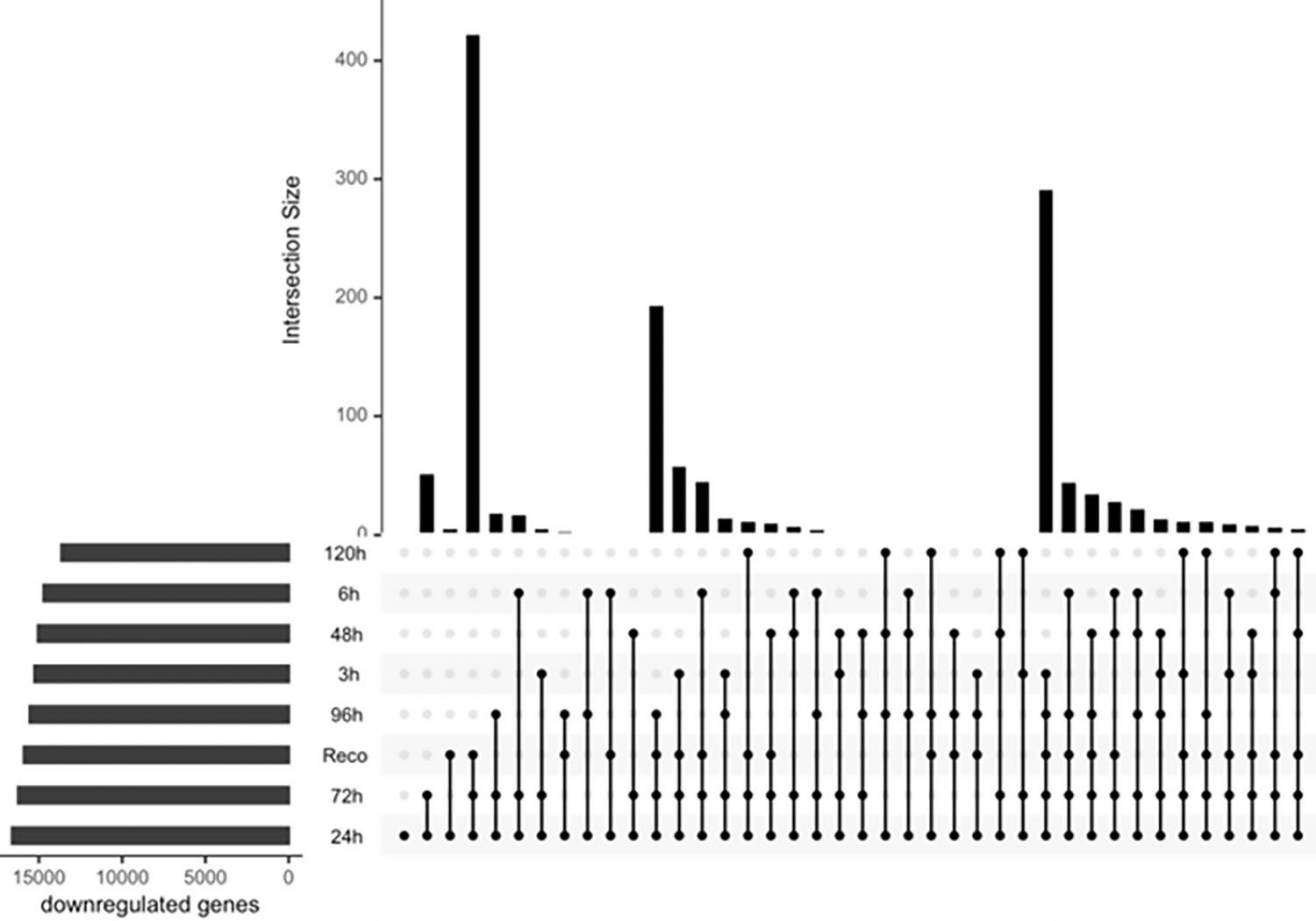
Upset plot showing differentially expressed downregulated genes under drought stress in different time series data. Vertical bars show the unique downregulated genes per time point. Horizontal bars display the total number of downregulated genes per time point. Dots connecting time points denote the unique downregulated genes to respective time points.

The DEGs were compared with their individual controls, and the analysis revealed interesting patterns (Figs. 3 and 4). In this study, we classified the 3h and 6h time points as the early stage of drought, the 48h and 72h time points as the medium stage, and the 96h and 120h time points as the late stage. It was observed that the most upregulated genes were found in the later stages of stress, specifically at 120 h (Fig. 3). This finding suggests that the cellular stress response becomes more pronounced as the duration of the stress increases. Interestingly, some genes were also found to be upregulated in the early stages of stress and overlapped with those found in the later stages. This observation implies that the cellular stress response may involve immediate and delayed mechanisms. And in the medium stages of water deficit, a few genes were found to be significantly upregulated. Although the number of DEGs was lower in this stage, it is important to note that these genes may play important roles in the early stages of the stress response. This study provides valuable insights into the temporal dynamics of gene expression in response to stress. The identification of DEGs at different time points highlights the importance of considering the duration of stress when studying cellular responses. Additionally, the overlapping DEGs observed across different stages suggest that the cellular stress response involves a complex interplay of immediate and delayed mechanisms.

### Gene ontology enrichment analysis

**Figure 5:**
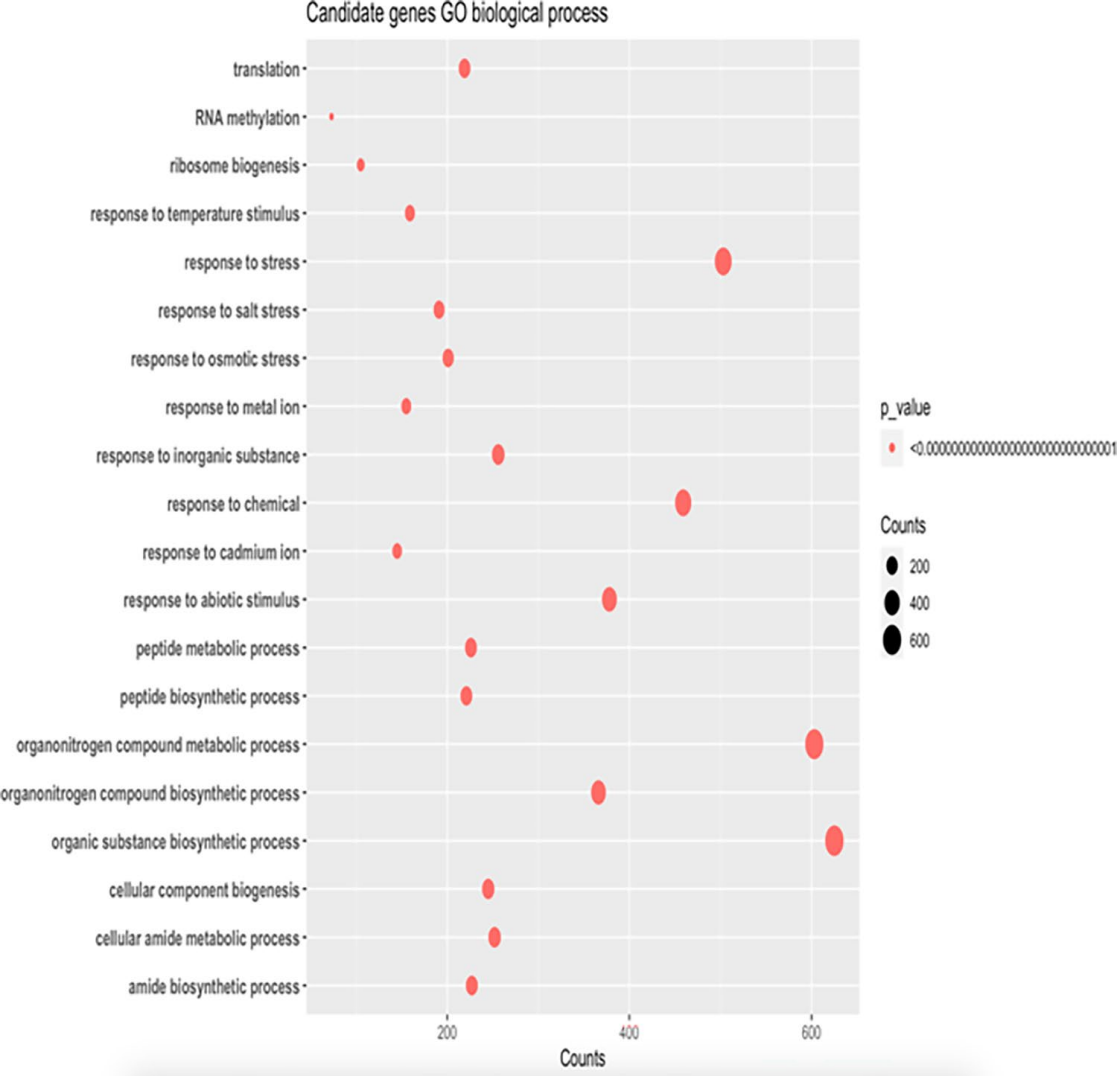
Functions of differentially expressed genes predicted from GO term of Tomato genome. X-axis shows the number of differentially expressed genes involved in the functions displayed in y-axis. The size of the points in the plot denotes the p-value.

Gene ontology enrichment analysis is a powerful tool used to interpret the biological function of differentially expressed genes (DEGs) in a high-throughput manner. In this study, the functions of DEGs were predicted from GO and the results were visualized in Fig. 5. The analysis revealed that a large proportion of DEGs (approximately 600 genes) were involved in organic substance biosynthetic processes and organonitrogen compounds. This finding suggests that the cellular stress response involves the production and modification of organic compounds, which play important roles in cellular metabolism and signaling pathways. Moreover, approximately 500 genes were found to be related to stress, indicating that the cellular stress response involves the activation of various stress response pathways. This finding is in agreement with previous studies that have shown that stress response genes are upregulated in response to various environmental stimuli. Additionally, more than 400 genes were found to respond to chemicals. These genes may be involved in the synthesis, transport, and degradation of chemical signals, or they may play roles in downstream signaling pathways that are activated by chemical stimuli. Furthermore, approximately 200 genes were found to be related to translation and responded to osmotic stress. This finding suggests that osmotic stress may affect translation processes, which play important roles in the synthesis of proteins that are involved in cellular responses to stress. In summary, the gene ontology enrichment analysis conducted in this study provides valuable insights into the biological function of DEGs in response to stress. The identification of genes involved in various metabolic, signaling, and stress response pathways highlights the complexity of the cellular stress response and emphasizes the importance of considering multiple biological processes when studying stress responses.

### Machine learning performance

**Figure 6:**
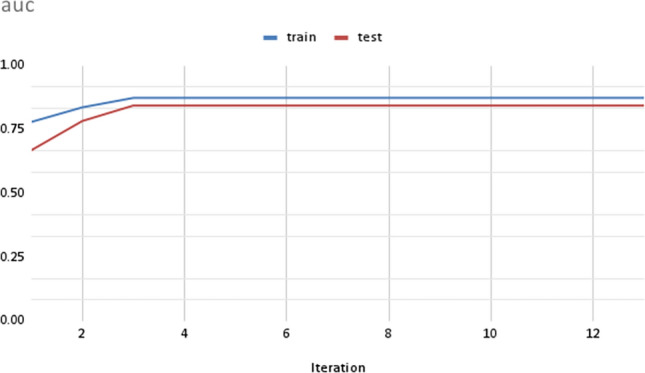
Plot showing the “auc” value for training data and test data sets over iterations.

In this study, we utilized gene expression values as features to train classification models using the XGBoost in R. XGBoost is a popular implementation of the gradient boosting algorithm, which combines multiple weak learners, such as shallow trees, into a strong one. To evaluate the performance of our models, we split our dataset into training and test sets. The accuracy of the models was measured using the area under the curve (“auc”) value, which is a commonly used metric for evaluating classification models. The results of our analysis, shown in Fig. 6, demonstrate that the accuracy of the training and test sets are quite close to each other, indicating that our models are well-fitted. Furthermore, we observed that the accuracy of the models increased over iterations, suggesting that the models learned from the data and were able to improve their performance as they were trained with more data. The use of the XGBoost in our study is particularly beneficial due to its ability to handle high-dimensional datasets, such as those generated from gene expression studies. By incorporating multiple weak learners, the algorithm can effectively capture the complex relationships between gene expression values and biological outcomes, such as disease states or cellular responses to stress. In summary, our results demonstrate the effectiveness of XGBoost in classifying biological samples based on gene expression values. The close agreement between the accuracies of the training and test sets suggests that our models are robust and generalizable. The use of XGBoost has enabled us to extract meaningful insights from high-dimensional gene expression datasets, and its flexibility makes it a valuable tool for future studies in this area.

### Feature importance

**Figure 7:**
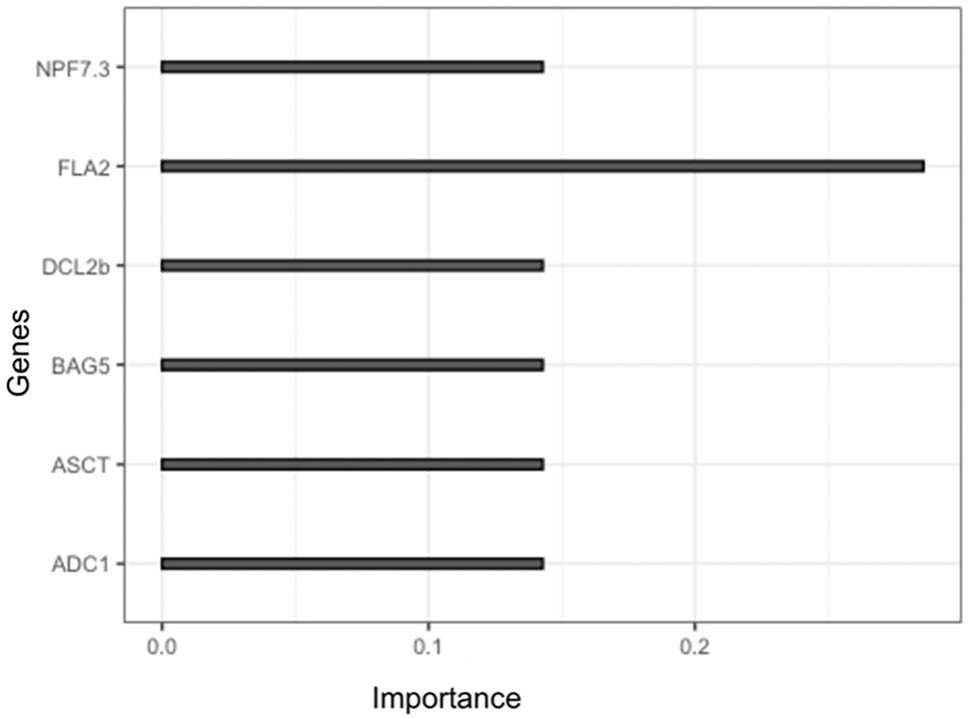
Candidate genes and their importance scores for drought response. The importance scores were obtained from machine learning models.

In this study, we employed a robust approach to determine the most influential genes that contribute to the classification of samples based on their gene expression values. Following the training and validation of our classification model using the XGBoost algorithm, we extracted the features with their corresponding importance scores. The importance score of a feature reflects its contribution to the classification of samples and thus provides valuable insights into the underlying biological mechanisms. Among the top six genes identified as candidate genes, we found that *FLA2* gene had the highest importance score (Fig. 7). This observation suggests that the expression of *FLA2* gene is a critical factor in determining the classification of samples in our study. In this context, classification entails the distinction between two classes: watered samples labeled as 0 and non-watered samples labeled as 1. These classes were defined as binary categories (0 and 1) within the model, and they were treated as the response variables. *FLA2* gene encodes for fasciclin-like arabinogalactan protein 2, which is a cell surface protein involved in various cellular processes such as cell adhesion, growth, and differentiation. The high importance score of *FLA2* gene in our analysis may indicate its involvement in the cellular stress response and thus may have potential implications in the development of stress-tolerant crops. It is worth noting that the other candidate genes identified in our analysis also play important roles in various cellular processes. Further investigation of these genes could provide valuable insights into the underlying biological mechanisms involved in stress responses. In conclusion, the identification of *FLA2* gene as the most important gene in our study highlights its potential significance in stress responses. The use of the XGBoost algorithm has enabled us to extract meaningful insights from high-dimensional gene expression datasets, and its flexibility makes it a valuable tool for future studies in this area.

### Gene association networks

**Figure 8:**
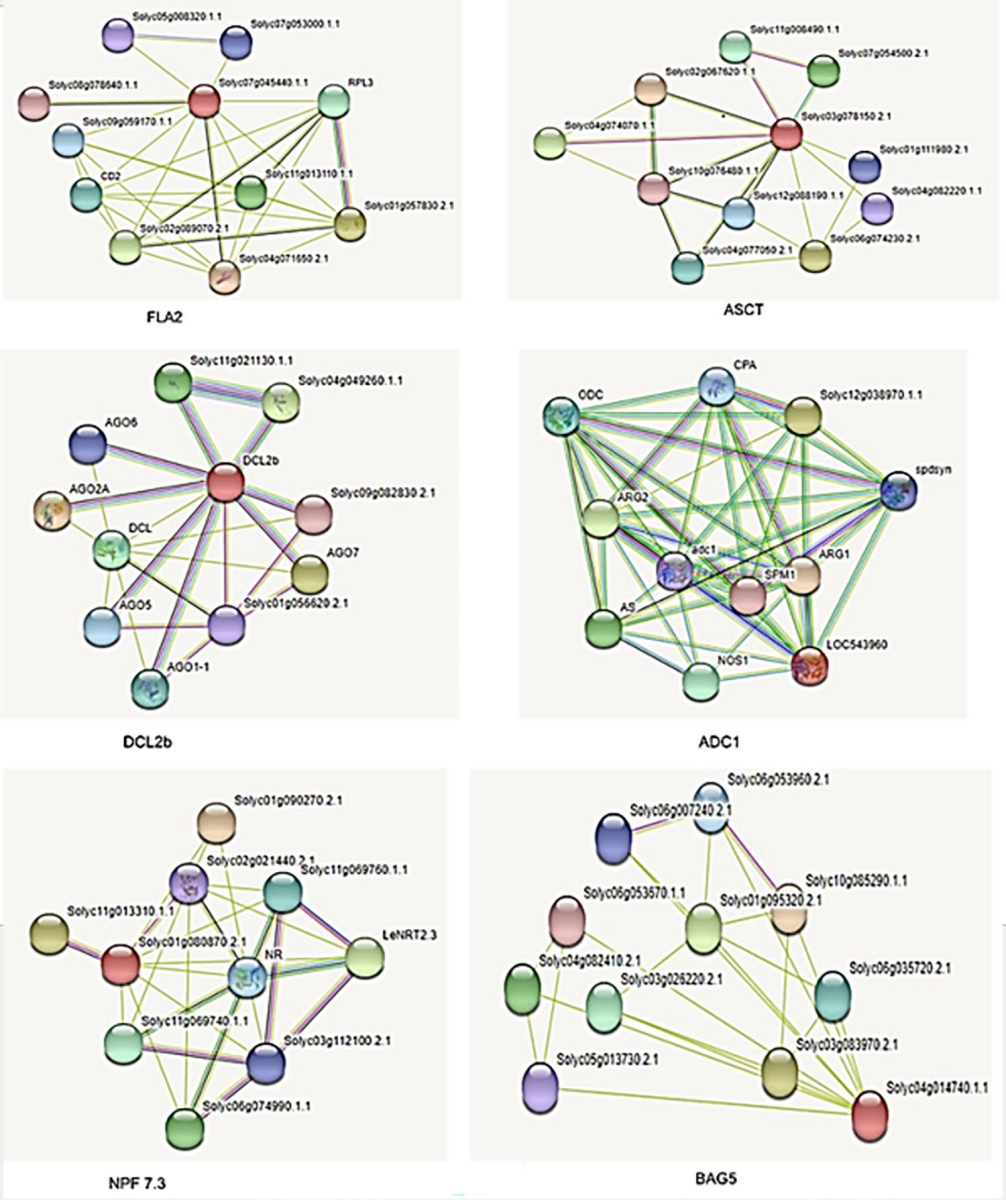
Interaction network for candidate genes. The red color nodes in the network correspond to the protein of the respective candidate genes. Purple edges signify experimentally determined interactions, turquoise edges indicate information extracted from curated databases. Green edges represent neighborhood genes, red edges denote gene fusion, dark blue edges signify gene co-occurrence, light green edges are indicative of text mining, black edges indicate co-expression between nodes, and light blue edges signify protein homology.

To gain a better understanding of the biological functions and interactions of the candidate genes identified in our study, we constructed gene association networks for each gene using publicly available databases. The resulting network for each candidate gene is shown in Fig. 8, with nodes representing genes and edges representing interactions between genes. In these networks, the high importance of candidate genes identified in our study within the network is highlighted by the colour red. We also observed that the interactors of these candidate genes showed drought stress-related functions, suggesting their involvement in the abiotic stress response. The gene association networks provide valuable insights into the potential pathways and mechanisms involved in the cellular stress response. By identifying the interactions between genes, we can better understand the complex interactions and functions of genes in the network. This information could be further used to develop targeted interventions for improving drought stress tolerance in crops.

Overall, the gene association networks of the candidate genes identified in our study highlight their potential significance in drought stress responses and provide a foundation for further investigation into their roles in drought-stress tolerance (Supplementary 2).

### Candidate genes overlapped with drought QTLs of Tomato

In this study, we aimed to explore the potential role of three candidate genes, *FLA2*, *ASCT*, and *NPF7.3*, in the response of tomato plants to drought stress. To investigate this, we compared the location of these genes with the previously identified QTLs associated with drought-related traits in tomato^17^.

Our analysis revealed that all three candidate genes overlapped with QTLs that were previously associated with drought stress, suggesting their potential involvement in the plant's response to water deficit conditions (Table 1). Specifically, the QTLs RIP, SSC, NFr, and FW were identified under drought stress for the traits of time to ripe, soluble solid content, number of fruits, and fruit weight, respectively.

**Table 1:** Drought stress QTLs and overlapped candidate genes.

QTLs	Physical Map (Mbp)	Gene	Transcripts
RIP3.1, SSC4.1, RIP1.1, NFr1.1	58.6651	FLA2	Solyc07g045440.1.1
FW2.2, NFr2.2, SSC11.1, RIP3.1	51.555	ASCT	Solyc03g078150.3.1
SSC1.1, NFr1.1	80.0395	NPF7.3	Solyc01g080870.3.1

The importance of our findings lies in the potential for identifying specific genes that contribute to the drought stress response of tomato plants. A better understanding of the genetic mechanisms involved in this response is crucial for developing crop varieties that can withstand environmental stressors such as water scarcity.

Our study suggests that *FLA2*, *ASCT*, and *NPF7.3* could be promising candidate genes for further investigation in the context of drought stress in tomato plants. Future studies could focus on elucidating the specific molecular pathways through which these genes affect the plant's response to water deficit conditions. This could lead to the development of more targeted breeding programs that utilize these genes to develop drought-tolerant tomato varieties.

## Discussion

The abstract of our study highlights the crucial importance of gaining a thorough understanding of the genetic composition of tomato plants to improve their ability to withstand drought stress, especially given the significant global demand for this staple crop. To achieve this objective, we employed state-of-the-art machine learning techniques to identify the most responsive genes associated with prolonged water scarcity in tomato plants from publicly available drought stress transcriptomics data in Tomato. Our investigation uncovered six promising candidate genes, with *FLA2* emerging as the top gene, indicating its potential as a critical target for enhancing drought resistance in tomatoes. Moreover, the gene ontology enrichment analysis conducted in our study demonstrated that the functions of the identified candidate genes were closely linked to drought stress. These findings are consistent with previous research^10^ reinforcing the notion that these candidate genes play a pivotal role in imparting drought tolerance in tomato plants. Our study significantly advances our knowledge of the molecular mechanisms underlying the response of tomato plants to drought stress. The identification of *FLA2* and other candidate genes, coupled with the functional analysis of their gene ontology, lays a robust foundation for future research focused on developing drought-resistant tomato varieties. Such research can help mitigate the negative impact of water scarcity on global food production, making our study highly pertinent and timely.

The findings of this study demonstrate that specific genes were upregulated in later stages of stress, whereas others overlapped with early stages, aligning with previous research on plant response to drought stress^18,19^. These results underscore the complexity of the molecular mechanisms involved in plant responses to stress and emphasize the need for a comprehensive understanding of these mechanisms that encompass multiple genetic pathways. By shedding light on the intricate mechanisms that govern plant response to drought stress, this study contributes to advancing our knowledge in the field and lays a foundation for future research aimed at developing drought-tolerant plant varieties.

This article emphasizes the critical importance of identifying candidate genes involved in the response to abiotic stress, particularly drought stress, and understanding their interactions using interaction networks. Our study focused on six genes (*FLA2*, *ASCT*, *DCL2b*, *ADC1*, *NPF7.3*, and *BAG5*), and the constructed interaction networks offer valuable insights into the potential pathways and mechanisms involved in drought responses. This research provides a foundation for developing targeted interventions to enhance stress tolerance in crops. The study highlights the significant role of the identified candidate genes in the response to abiotic stress, especially drought stress, with these genes exhibiting functions related to the drought stress response could lead to cross-talk of other abiotic stresses. Our findings are consistent with previous research^20^, in which various genes were identified, including SlMAPK3, a gene involved in multiple stress responses in tomato. The over-expression of SlMAPK3 improved the tolerance of tomato plants to drought stress by regulating the expression of genes involved in drought stress responses. Therefore, our study makes a valuable contribution to ongoing research on drought stress tolerance in tomato. The identification of candidate genes involved in drought stress response and the construction of interaction networks lay a foundation for further investigation into the molecular mechanisms involved in stress tolerance. These findings could be leveraged to develop new strategies for improving crop productivity and sustainability in the face of drought stress and other environmental stressors, ultimately benefiting global food security^11,15,17,21^.

The integration of bioinformatics and computational methods, particularly machine learning, has greatly advanced the field of plant stress response research. The study presented in this paper is a remarkable example of how such approaches can provide novel insights into the genetic mechanisms underlying plant responses to drought stress^22^. By identifying candidate genes and regulatory elements involved in stress response, machine learning can help develop crop breeding programs that enhance drought stress tolerance, ultimately contributing to addressing food security challenges^13,21^. The use of machine learning allows for the analysis of large amounts of genomic data and the identification of complex gene networks involved in stress response. This approach has enabled researchers to identify critical genetic targets and pathways that can be manipulated to enhance stress tolerance in crops. Such insights can be leveraged to develop crop varieties that can withstand harsh environmental conditions, including drought stress, which is one of the most significant challenges facing global food production^13,19^. Furthermore, machine learning can also help identify and prioritize the most promising candidate genes and regulatory elements for further experimental validation. This approach can significantly accelerate the breeding process by reducing the time and resources required to develop new crop varieties. Therefore, the integration of bioinformatics and computational methods, particularly machine learning, represents a powerful approach to identifying the genetic mechanisms underlying plant responses to environmental stress.

Such approaches have the potential to make significant contributions to addressing food security challenges, particularly in areas where drought stress is a significant constraint on crop production. The use of such methods can ultimately lead to the development of drought-resistant crop varieties, which is critical for ensuring global food security in the face of climate change and other environmental challenges. Therefore, the integration of bioinformatics and computational methods is a promising tool for future research aimed at developing crops that can withstand environmental stressors and ultimately improve global food production.

## Conclusion

In this study, we demonstrated that some genes of tomato were significantly upregulated in later stages of water stress, whereas some other genes were also found in the early stages and overlapped with later stages. Our results suggest that some genes play distinct roles in the plant's response to drought stress. Therefore, a comprehensive understanding of these genes is crucial for developing strategies to improve crop resistance to stress. Furthermore, our machine learning approach resulted in identification of six candidate genes, with *FLA2* having the highest importance score, provides a starting point for further research into the specific functions and mechanisms of the genes in the context of drought stress. The gene ontology enrichment analysis predicted that the functions of candidate genes are related to drought stress, indicating that they may play crucial roles in the plant's ability to cope with environmental stress. Our study suggests that *FLA2*, *ASCT*, and *NPF7.3* could be promising candidate genes for further investigation in the context of drought stress in tomato plants. Overall, this study highlights the importance of understanding the genetic makeup of tomato plants to develop strategies to increase stress resistance and fruit quality. The findings of this study shed light on the genetic makeup of tomato plants and their response to prolonged water deficit, and environmental stress that can significantly impact crop yield and quality.

## Methods and materials

### RNA seq data

For drought stress, RNA Seq data of tomatoes were extracted from Gene Expression Omnibus (GEO) (https://www.ncbi.nlm.nih.gov/geo/). The data was collected using the following keywords in GEO: "*Solanum lycopersicum*"[Organism] OR Tomato [All Fields]) AND ("drought response"[MeSH Terms] OR Drought stress[All Fields]) (Fig. 1). RNA Seq data were downloaded by SRAToolkit. Each treatment has its corresponding controls. Data were sorted according to time series points of 3, 6, 48, 72, 96, and 120 h of treatment respectively. There was also data for recovery samples (Supplementary 1).

### Mapping

Reads were mapped against the coding sequence (CDS) of the Heinz 1706 genome. The latest annotation (ITAG4.1, https://solgenomics.net/) of the Heinz 1706 genome was used. Kallisto was used for mapping. 34688 genes were found after mapping. The abundance file contained transcript id, length, effective length, estimated counts, and TPM (Transcript per million). Transcript id estimated counts and TPM values of all samples were gathered in one tab-separated value file for further analysis. The estimated counts of all samples per time point were averaged and labeled as a single time point. This way we harmonized respective samples per time points.

### Gene expression analysis

Differentially expressed genes (DEGs) were analyzed using the Bioconductor edgeR package in R. Estimated counts value of all samples were taken to analyze differentially expressed genes. Data were normalized with library size. Dispersions of samples were calculated with glm approaches of the edgeR package by comparing the treatments with their respective controls. Exact tests were carried out to verify the significance of change gene expression and their log fold change values. Multiple testing corrections were done with the topTags function. It ranks genes by p-value. The threshold of considering differentially expressed genes was False discovery rate (FDR) < 0.01. For upregulated genes, the logFC threshold was > 1, and for downregulated genes, it was < −1. Visualizing of gene expression was done with heatmap using the Complexheatmap Bioconductor package of R. Gene expression was shown with genotype-specific, tissue-specific, stress-specific, heat level-specific, and sample-specific using row Annotation, bottom Annotation, and top Annotation functions of Complex heatmap package in R. Intersection and total set of genes of samples was visualized with an upset plot using R for both upregulated genes and downregulated genes. Some selected transcription factors from diversified families were collected in literature which are said to be drought-responsive genes. The expressions of that transcription factor were also analyzed across time series scales by comparing them with their respective controls.

### Gene ontology analysis

Gene ontology enrichment analysis of significant genes of samples was carried out with the topGO package in R. The genome annotation format (GAF, ITAG4.1) file of tomato was collected from (https://doi.org/10.25739/zh2v-4p15) and a map file was created with the bash script. The map file contained two columns with tabs separated where the first column was GO terms and the second column was genes to respective GO terms with space-separated. The functions were considered only those who were involved in biological processes. Fisher's classic test was done to find significant levels of GO terms and their functions. The top 15 functions were selected as thresholds.

### Candidate selection

We used a tree model with gradient boosting, XGBoost in R to train and test the models. The estimated counts values of samples were normalized by their library sizes. The variance stabilized matrix was calculated after differentially expressed genes were identified. The genes were filtered out when their variance were below 95%. After the filtering and normalization, 1735 genes were considered for machine learning model as features for 159 samples. We split the data randomly into training (80%) and testing (20%) sets. We used five-fold internal cross-validation to select the optimized hyperparameters. The data aimed for binary classification, where the watered samples were labeled as 0, and the non-watered samples were labeled as 1. We tuned “nrounds” (number of trees), “colsample_bytree” (the proportion of features for constructing each tree), “subsamples” (the portion of training data samples for training each additional tree), and “eta” (shrinkage of feature weights to make the boosting process more conservative and prevent overfitting) in an XGBoost: classification model. We used the XGBoost-generated feature importance score that indicates how useful each feature was in the construction of the model.

### Interaction network

To find out the likely interactions of candidate genes the STRING ((https://string-db.org) tool was used^23^. For the search interface, parameters were set to full network type, confidence score > 0.4, and more than 10 interactors. In the organism interface, it was restricted to *Solanum lycopersicum*. In the networks graph, the colored nodes display the proteins and the edges represent the interactions.

### Supplementary Information

Supplementary Information 1.

Supplementary Information 2.

## Data Availability

The source of all data analysed during this study are included in the supplementary files. The codes for the current study available from the corresponding authors on reasonable request.
