# Supplementary material for: Drought-responsive genes in tomato: meta-analysis of gene expression using machine learning
Source: Sci Rep. 2023 Nov 8;13:19374. doi: 10.1038/s41598-023-45942-2 (PMC10632472; doi:10.1038/s41598-023-45942-2)
Supplement: Supplementary file 1 — Supplementary Information 1. [file 41598_2023_45942_MOESM1_ESM.pdf]

Drought-Responsive Genes in Tomato: Meta-Analysis of Gene Expression Using Machine Learning

Rabiul Haq Chowdhury1† & Fatiha Sultana Eti2†, Rayhan Ahmed1 , Shipan Das Gupta3, Pijush Kanti Jhan1, Tofazzal Islam4, Md. Atiqur Rahman Bhuiyan1 , Mehede Hassan Rubel1* and Abul Khayer2*‡

correspondence should be addressed at **zitunstu24@gmail.com (Abul Khayer)**

Supplementary table 1: SRA Accession table

sra_run_id	layout	fragment_length_average	fragment_length_sd	publication_id	species	genotype.name	tissue	stress_type	stress.duration	sample.group	respective_control
SRR11855627	paired			p20	*Solanum lycopersicum*	M82	leaf	none	0	e20-0	
SRR11855628	paired			p20	*Solanum lycopersicum*	M82	leaf	none	0	e20-0	
SRR11855629	paired			p20	*Solanum lycopersicum*	M82	leaf	none	0	e20-0	
SRR11855630	paired			p20	*Solanum lycopersicum*	M82	leaf	drought	24	e20-1	e20-0
SRR11855631	paired			p20	*Solanum lycopersicum*	M82	leaf	drought	24	e20-1	e20-0
SRR11855632	paired			p20	*Solanum lycopersicum*	M82	leaf	drought	24	e20-1	e20-0
SRR11855633	paired			p20	*Solanum lycopersicum*	M82	leaf	drought	48	e20-2	e20-0
SRR11855634	paired			p20	*Solanum lycopersicum*	M82	leaf	drought	48	e20-2	e20-0
SRR11855635	paired			p20	*Solanum lycopersicum*	M82	leaf	drought	48	e20-2	e20-0
SRR11855636	paired			p20	*Solanum lycopersicum*	M82	leaf	drought	72	e20-3	e20-0
SRR11855637	paired			p20	*Solanum lycopersicum*	M82	leaf	drought	72	e20-3	e20-0
SRR11855638	paired			p20	*Solanum lycopersicum*	M82	leaf	drought	72	e20-3	e20-0
SRR11855639	paired			p20	*Solanum lycopersicum*	M82	leaf	drought	96	e20-4	e20-0
SRR11855640	paired			p20	*Solanum lycopersicum*	M82	leaf	drought	96	e20-4	e20-0
SRR11855641	paired			p20	*Solanum lycopersicum*	M82	leaf	drought	96	e20-4	e20-0
SRR11855642	paired			p20	*Solanum lycopersicum*	M82	leaf	drought	120	e20-5	e20-0
SRR11855643	paired			p20	*Solanum lycopersicum*	M82	leaf	drought	120	e20-5	e20-0
SRR11855644	paired			p20	*Solanum lycopersicum*	M82	leaf	drought	120	e20-5	e20-0
SRR11855645	paired			p20	*Solanum lycopersicum*	M82	leaf	drought	recovery	e20-6	e20-0
SRR11855646	paired			p20	*Solanum lycopersicum*	M82	leaf	drought	recovery	e20-6	e20-0
SRR11855647	paired			p20	*Solanum lycopersicum*	M82	leaf	drought	recovery	e20-6	e20-0
SRR11539290	paired			p22	*Solanum lycopersicum*	Micro tom	leaf	none	0	e22-1-0	
SRR11539291	paired			p22	*Solanum lycopersicum*	Micro tom	leaf	none	0	e22-1-0	
SRR11539292	paired			p22	*Solanum lycopersicum*	Micro tom	leaf	none	0	e22-1-0	
SRR11539293	paired			p22	*Solanum lycopersicum*	Micro tom	leaf	drought	3	e22-1	e22-1-0
SRR11539294	paired			p22	*Solanum lycopersicum*	Micro tom	leaf	drought	3	e22-1	e22-1-0
SRR11539295	paired			p22	*Solanum lycopersicum*	Micro tom	leaf	drought	3	e22-1	e22-1-0
SRR11539296	paired			p22	*Solanum lycopersicum*	Micro tom	leaf	drought	6	e22-2	e22-1-0
SRR11539297	paired			p22	*Solanum lycopersicum*	Micro tom	leaf	drought	6	e22-2	e22-1-0
SRR11539298	paired			p22	*Solanum lycopersicum*	Micro tom	leaf	drought	6	e22-2	e22-1-0
SRR11539299	paired			p22	*Solanum lycopersicum*	Micro tom	leaf	none	0	e22-3-0	
SRR11539300	paired			p22	*Solanum lycopersicum*	Micro tom	leaf	none	0	e22-3-0	
SRR11539301	paired			p22	*Solanum lycopersicum*	Micro tom	leaf	none	0	e22-3-0	
SRR11539302	paired			p22	*Solanum lycopersicum*	Micro tom	leaf	drought	3	e22-3	e22-3-0
SRR11539303	paired			p22	*Solanum lycopersicum*	Micro tom	leaf	drought	3	e22-3	e22-3-0
SRR11539304	paired			p22	*Solanum lycopersicum*	Micro tom	leaf	drought	3	e22-3	e22-3-0
SRR11539305	paired			p22	*Solanum lycopersicum*	Micro tom	leaf	drought	6	e22-4	e22-3-0
SRR11539306	paired			p22	*Solanum lycopersicum*	Micro tom	leaf	drought	6	e22-4	e22-3-0
SRR11539307	paired			p22	*Solanum lycopersicum*	Micro tom	leaf	drought	6	e22-4	e22-3-0
SRR11829574	paired			p23	*Solanum lycopersicum*	M82	leaf	none	0	e23-0	
SRR11829573	paired			p23	*Solanum lycopersicum*	M82	leaf	none	0	e23-0	
SRR11829562	paired			p23	*Solanum lycopersicum*	M82	leaf	none	0	e23-0	
SRR11829551	paired			p23	*Solanum lycopersicum*	M82	leaf	drought	24	e23-1	e23-0
SRR11829541	paired			p23	*Solanum lycopersicum*	M82	leaf	drought	24	e23-1	e23-0
SRR11829540	paired			p23	*Solanum lycopersicum*	M82	leaf	drought	24	e23-1	e23-0
SRR11829539	paired			p23	*Solanum lycopersicum*	M82	leaf	drought	48	e23-2	e23-0
SRR11829538	paired			p23	*Solanum lycopersicum*	M82	leaf	drought	48	e23-2	e23-0
SRR11829537	paired			p23	*Solanum lycopersicum*	M82	leaf	drought	48	e23-2	e23-0
SRR11829536	paired			p23	*Solanum lycopersicum*	M82	leaf	drought	72	e23-3	e23-0
SRR11829572	paired			p23	*Solanum lycopersicum*	M82	leaf	drought	72	e23-3	e23-0
SRR11829571	paired			p23	*Solanum lycopersicum*	M82	leaf	drought	72	e23-3	e23-0
SRR11829570	paired			p23	*Solanum lycopersicum*	M82	leaf	drought	96	e23-4	e23-0
SRR11829569	paired			p23	*Solanum lycopersicum*	M82	leaf	drought	96	e23-4	e23-0
SRR11829568	paired			p23	*Solanum lycopersicum*	M82	leaf	drought	96	e23-4	e23-0
SRR11829567	paired			p23	*Solanum lycopersicum*	M82	leaf	drought	120	e23-5	e23-0
SRR11829566	paired			p23	*Solanum lycopersicum*	M82	leaf	drought	120	e23-5	e23-0
SRR11829565	paired			p23	*Solanum lycopersicum*	M82	leaf	drought	120	e23-5	e23-0
SRR11829564	paired			p23	*Solanum lycopersicum*	M82	leaf	drought	recovery	e23-6	e23-0
SRR11829563	paired			p23	*Solanum lycopersicum*	M82	leaf	drought	recovery	e23-6	e23-0
SRR11829561	paired			p23	*Solanum lycopersicum*	M82	leaf	drought	recovery	e23-6	e23-0

Supplementary table 2: Publication links

p20	https://doi.org/10.3390/ijms22031387
p22	https://doi.org/10.3389/fgene.2020.00540
p23	https://doi.org/10.3390/ijms22031387

Supplementary table 3: Description of the candidate genes

transcripts	gene	description
Solyc07g045440.1.1	FLA2	Fasciclin-like arabinogalactan protein 2
Solyc03g078150.3.1	ASCT	Amino acid transporter family protein
Solyc10g054440.3.1	ADC1	arginine decarboxylase 1
Solyc01g080870.3.1	NPF7.3	Protein NRT1/ PTR FAMILY 7.3
Solyc06g072430.3.1	BAG5	BAG family molecular chaperone regulator 5
Solyc11g008540.3.1	DCL2b	Dicer-like 2b
