# Supplementary material for: Drought-responsive genes in tomato: meta-analysis of gene expression using machine learning
Source: Sci Rep. 2023 Nov 8;13:19374. doi: 10.1038/s41598-023-45942-2 (PMC10632472; doi:10.1038/s41598-023-45942-2)
Supplement: Supplementary file 2 — Supplementary Information 2. [file 41598_2023_45942_MOESM2_ESM.pdf]

Drought-Responsive Genes in Tomato: Meta-Analysis of Gene Expression Using Machine Learning

Rabiul Haq Chowdhury1† & Fatiha Sultana Eti2†, Rayhan Ahmed1 , Shipan Das Gupta3, Pijush Kanti Jhan1, Tofazzal Islam4, Md. Atiqur Rahman Bhuiyan1 , Mehede Hassan Rubel1* and Abul Khayer2*‡

correspondence should be addressed at **zitunstu24@gmail.com (Abul Khayer)**

Supplementary table 4: Descriptions of the columns for the Supplementary table 5

column	description
node	name of protein connection. Bold node is candidate identifiers
identifier	organism code and protein ids
domain_summary	domain description
annotation	source
other_names	names according to function or position

Supplementary table 5: Interactors of the Candidate genes and their descriptions

nodes of FLA2	identifier	domain_summary	annotation	other_names
RPL3	4081.Solyc01g104590.2.1	Large subunit ribosomal protein l3e	https://smart.embl.de/smart/DDvec.cgi?smart=389:Pfam_Ribosomal_L3(1|370)+	Large subunit ribosomal protein L3e,RPL3,Ribosomal protein L3,
Solyc01g057830.2.1	4081.Solyc01g057830.2.1	annotation not available	https://smart.embl.de/smart/DDvec.cgi?smart=412:S1(95|167)+S1(183|249)+S1(260|330)+	30S ribosomal protein S1,30S ribosomal protein S1, chloroplastic,Small subunit ribosomal protein S1
Solyc02g089070.2.1	4081.Solyc02g089070.2.1	Eukaryotic translation initiation factor 3 subunit M	https://smart.embl.de/smart/DDvec.cgi?smart=410:PINT(290|380)+	Translation initiation factor 3 subunit M,eIF3m,sly:101255548
Solyc04g071650.2.1	4081.Solyc04g071650.2.1	Cellulose synthase a catalytic subunit; Belongs to the glycosyltransferase 2 family.	https://smart.embl.de/smart/DDvec.cgi?smart=1090:RING(39|84)+Pfam_Glyco_trans_2_3(755|919)+	A0A3Q7G6I1_SOLLC,Cellulose synthase,Cellulose synthase A
**Solyc07g045440.1.1**	4081.Solyc07g045440.1.1	annotation not available	https://smart.embl.de/smart/DDvec.cgi?smart=409:SIGNAL(1|25)+FAS1(61|176)+FAS1(225|329)+	A0A3Q7HCZ8_SOLLC,Fasciclin-like arabinogalactan protein 2
Solyc11g013110.1.1	4081.Solyc11g013110.1.1	Flavonol synthase/flavanone 3-hydroxylase; Belongs to the iron/ascorbate-dependent oxidoreductase family	https://smart.embl.de/smart/DDvec.cgi?smart=352:Pfam_DIOX_N(57|173)+Pfam_2OG-FeII_Oxy(214|313)+	Fe2OG dioxygenase domain-containing protein,Flavonol synthase ,Flavonol synthase/flavanone 3-hydroxylase

nodes of ASCT	identifier	domain_summary	annotation	other_names
Solyc01g111980.2.1	4081.Solyc01g111980.2.1	annotation not available	https://smart.embl.de/smart/DDvec.cgi?smart=566:Pfam_Aa_trans(139|555)+	101250935,A0A3Q7EWB1,A0A3Q7EWB1_SOLLC,Aa_trans domain-containing protein,K4B460,K4B460_SOLLC,LOC101250935,Lysine histidine transporter-like 8,Solyc01g111980.2,Solyc01g111980.2.1,UPI0002767ABA,Uncharacterized protein,XM_004231029,XM_004231029.1,XM_004231029.2,XP_004231077,XP_004231077.1,sly:101250935
Solyc02g067620.1.1	4081.Solyc02g067620.1.1	Solute carrier family 30 (zinc transporter); Cation efflux family protein	https://smart.embl.de/smart/DDvec.cgi?smart=902:TRANS(70|92)+TRANS(102|124)+TRANS(207|229)+TRANS(249|271)+TRANS(292|314)+TRANS(371|393)+Pfam_Cation_efflux(434|819)+	101260896,Cation efflux family protein,K4B794,K4B794_SOLLC,LOC101260896,Solyc02g067620.1,Solyc02g067620.1.1,UPI0002767587,Uncharacterized protein,Uncharacterized protein LOC101260896,XM_004233290,XM_004233290.1,XP_004233338,XP_004233338.1,sly:101260896
**Solyc03g078150.2.1**	4081.Solyc03g078150.2.1	Solute carrier family 32 (vesicular inhibitory amino acid transporter)	https://smart.embl.de/smart/DDvec.cgi?smart=525:Pfam_Aa_trans(136|519)+	101267002,A0A3Q7G8X4,A0A3Q7G8X4_SOLLC,Aa_trans domain-containing protein,Amino acid transporter AVT1A isoform X1,K4BHP5,K4BHP5_SOLLC,LOC101267002,Solyc03g078150.2,Solyc03g078150.2.1,UPI0002767DC2,Uncharacterized protein,XM_004235028,XM_004235028.1,XM_004235028.3,XP_004235076,XP_004235076.1,sly:101267002
Solyc04g074070.1.1	4081.Solyc04g074070.1.1	Mfs transporter, Major facilitator superfamily protein; facilitator superfamily. Sugar transporter family	https://smart.embl.de/smart/DDvec.cgi?smart=509:Pfam_MFS_1(32|440)+	101267019,A0A3Q7G7B4,A0A3Q7G7B4_SOLLC,K4BTU5,K4BTU5_SOLLC,LOC101267019,MFS domain-containing protein,MFS transporter, SP family, sugar:H+ symporter,Major facilitator superfamily protein,Solyc04g074070.1,Solyc04g074070.1.1,Sugar transport protein 5
Solyc04g077050.2.1	4081.Solyc04g077050.2.1	annotation not available	https://smart.embl.de/smart/DDvec.cgi?smart=481:Pfam_Aa_trans(33|469)+	101245454,A0A3Q7G8A7,A0A3Q7G8A7_SOLLC,Aa_trans domain-containing protein,Amino acid permease 6,K4BUE2,K4BUE2_SOLLC,Solyc04g077050.2,Solyc04g077050.2.1,UPI0002766250,Uncharacterized protein,XM_010321942.2,XP_010320244,XP_010320244.2,sly:101245454
Solyc04g082220.1.1	4081.Solyc04g082220.1.1	annotation not available	https://smart.embl.de/smart/DDvec.cgi?smart=458:Pfam_Aa_trans(87|457)+	101251882,A0A3Q7G8H4,A0A3Q7G8H4_SOLLC,Aa_trans domain-containing protein,K4BVU9,K4BVU9_SOLLC,LOC101251882,Lysine histidine transporter-like 8,Solyc04g082220.1,Solyc04g082220.1.1,UPI0002766490,Uncharacterized protein,XM_004238579,XM_004238579.1,XM_004238579.3,XP_004238627,XP_004238627.1,XP_004238627.2,sly:101251882
Solyc06g074230.2.1	4081.Solyc06g074230.2.1	Belongs to the Casparian strip membrane proteins (CASP) family	https://smart.embl.de/smart/DDvec.cgi?smart=210:Pfam_DUF588(46|195)+	101263718,A0A3Q7H363,A0A3Q7H363_SOLLC,CASP-like protein,Casparian strip membrane protein 3-like,K4C9E1,K4C9E1_SOLLC,LOC101263718,Solyc06g074230.2,Solyc06g074230.2.1,UPI00027663BD,XM_004242063,XM_004242063.1,XM_004242063.3,XP_004242111,XP_004242111.1,sly:101263718
Solyc07g054500.2.1	4081.Solyc07g054500.2.1	annotation not available	https://smart.embl.de/smart/DDvec.cgi?smart=791:SIGNAL(1|24)+TRANS(36|58)+LRR(96|123)+LRR(142|165)+LRR(166|189)+LRR(190|212)+TRANS(322|344)+Pfam_Pkinase_Tyr(502|776)+	101259169,A0A3Q7HCW8,A0A3Q7HCW8_SOLLC,K4CFZ5,K4CFZ5_SOLLC,LOC101259169,LRRNT_2 domain-containing protein,Solyc07g054500.2,Solyc07g054500.2.1,UPI000276C5EC
Solyc10g076480.1.1	4081.Solyc10g076480.1.1	Ammonium transporter, Ammonium transporter 2	https://smart.embl.de/smart/DDvec.cgi?smart=483:Pfam_Ammonium_transp(25|450)+	101246876,A0A3Q7IIV2,A0A3Q7IIV2_SOLLC,ATAMT2,Ammonium transporter,Ammonium transporter 2,Ammonium transporter, Amt family,K4D1Z0,K4D1Z0_SOLLC,LOC101246876,Solyc10g076480.1,Solyc10g076480.1.1,ammonium transporter 2,sly:101246876
Solyc11g008490.1.1	4081.Solyc11g008490.1.1	Pumilio rna-binding family; Pumilio 4	https://smart.embl.de/smart/DDvec.cgi?smart=915:Pfam_NABP(443|571)+Pumilio(573|608)+Pumilio(609|644)+Pumilio(645|680)+Pumilio(681|716)+Pumilio(717|753)+Pumilio(754|789)+Pumilio(790|825)+Pumilio(832|867)+	101246586,A0A3Q7IQN8,A0A3Q7IQN8_SOLLC,APUM4,K4D5A9,K4D5A9_SOLLC,LOC101246586,PUM-HD domain-containing protein,Pumilio 4,Pumilio RNA-binding family,Pumilio homolog 4-like,Solyc11g008490.1,Solyc11g008490.1.1,UPI000276A8DC,pumilio 4,sly:101246586
Solyc12g088190.1.1	4081.Solyc12g088190.1.1	annotation not available	https://smart.embl.de/smart/DDvec.cgi?smart=488:Pfam_Aa_trans(38|474)+	101252966,A0A3Q7JCH6,A0A3Q7JCH6_SOLLC,Aa_trans domain-containing protein,Amino acid permease 6,K4DGP0,K4DGP0_SOLLC,Solyc12g088190.1,Solyc12g088190.1.1,UPI000276A9C4,Uncharacterized protein,XM_010316294.2,XP_010314596,XP_010314596.2,sly:101252966

nodes of ADC1	identifier	domain_summary	annotation	other_names
ARG1	4081.Solyc01g091160.2.1	Arginase 1 ; Belongs to the arginase family	https://smart.embl.de/smart/DDvec.cgi?smart=338:Pfam_Arginase(59|334)+	3.5.3.1,543944,AAV36808.1,AEB39503.1,ARG1,AY656837,Arginase ,Arginase 1,EC 3.5.3.1,F5BCX4_SOLLC,HQ844971,LeARG1,NM_001247649,NM_001247649.1,NP_001234578,NP_001234578.1,Q5UNS2,Q5UNS2_SOLLC,Solyc01g091160.2,Solyc01g091160.2.1,UPI000045426E,sly:543944
ARG2	4081.Solyc01g091170.2.1	Arginase 2 ; Belongs to the arginase family	https://smart.embl.de/smart/DDvec.cgi?smart=338:Pfam_Arginase(59|334)+	3.5.3.1,544271,A0A3Q7EK65,A0A3Q7EK65_SOLLC,AAV36809.1,ARG2,AY656838,Arginase ,Arginase 2,EC 3.5.3.1,LeARG2,NM_001246922,NM_001246922.1,NP_001233851,NP_001233851.1,Q5UNS1,Q5UNS1_SOLLC,Solyc01g091170.2,Solyc01g091170.2.1,UPI000045426F,Uncharacterized protein,XP_010324097.1,sly:544271
AS	4081.Solyc04g076320.2.1	Argininosuccinate lyase; L-Aspartase-like family protein	https://smart.embl.de/smart/DDvec.cgi?smart=525:Pfam_Lyase_1(70|364)+Pfam_ASL_C2(427|495)+	101255089,4.3.2.1,A0A3Q7H0R9,A0A3Q7H0R9_SOLLC,AS,Argininosuccinate lyase,Argininosuccinate lyase ,EC 4.3.2.1,K4BU70,K4BU70_SOLLC,L-Aspartase-like family protein,LOC101255089,NP_001316165,Solyc04g076320.2,Solyc04g076320.2.1,UPI0002765C72,Uncharacterized protein,XM_004237948,XM_004237948.1,XP_004237996,XP_004237996.1,sly:101255089
CPA	4081.Solyc11g068540.1.1	N-carbamoylputrescine amidase; Belongs to the carbon-nitrogen hydrolase superfamily	https://smart.embl.de/smart/DDvec.cgi?smart=300:Pfam_CN_hydrolase(9|274)+	3.5.1.53,544141,AGUB_SOLLC,CAB45873.1,CPA,EC 3.5.1.53,N-carbamoylputrescine amidase,N-carbamoylputrescine amidase ,NM_001247456,NM_001247456.1,NP_001234385,NP_001234385.1,Q9XGI9,Solyc11g068540.1,Solyc11g068540.1.1,UPA00534,UPI00000A5905,Y19104,sly:544141
LOC543960	4081.Solyc01g110440.2.1	Arginine decarboxylase ; Belongs to the Orn/Lys/Arg decarboxylase class-II family.	https://smart.embl.de/smart/DDvec.cgi?smart=607:Pfam_Orn_Arg_deC_N(12|287)+	4.1.1.19,543960,AAA61347.1,ADC,ADK91572.1,AEW69809.1,ARGDC,Arginine decarboxylase,Arginine decarboxylase ,E0ADH6_SOLLC,EC 4.1.1.19,G8Z275_SOLLC,GQ261253,HM629957,K4B3Q7_SOLLC,L16582,LOC543960,NP_001234649,P49726,SPE1_SOLLC,Solyc01g110440.2,Solyc01g110440.2.1,UPI0002768FA9,sly:543960
NOS1	4081.Solyc03g093240.2.1	Putative nitric oxide synthase;	https://smart.embl.de/smart/DDvec.cgi?smart=560:Pfam_MMR_HSR1(278|374)+	1.14.13.39,778272,A0A3Q7GAT5,A0A3Q7GAT5_SOLLC,ABG25037.1,ATNOS1,CP-type G domain-containing protein,DQ539436,EC 1.14.13.39,K4BIK1,K4BIK1_SOLLC,NOS1,Nitric oxide synthase,Nitric-oxide synthase, plant ,Putative nitric oxide synthase,Q157M9_SOLLC,Solyc03g093240.2,Solyc03g093240.2.1,UPI0002768EF3,Uncharacterized protein,XM_004235117,XM
ODC	4081.Solyc04g082030.1.1	Ornithine decarboxylase;	https://smart.embl.de/smart/DDvec.cgi?smart=431:Pfam_Orn_Arg_deC_N(70|304)+	4.1.1.17,544209,AAB82301.2,AAC61845.1,AF029349,AF030292,DCOR_SOLLC,EC 4.1.1.17,LEODC,LeODC,NM_001247687,NM_001247687.1,NP_001234616,NP_001234616.1,O22616,O82420,ODC,Ornithine decarboxylase,Ornithine decarboxylase ,Solyc04g082030.1,Solyc04g082030.1.1,UPI00000ACEAD,sly:544209
SPM1	4081.Solyc03g007240.2.1	Putative spermine synthase ; Belongs to the spermidine/spermine synthase family	https://smart.embl.de/smart/DDvec.cgi?smart=356:Pfam_Spermine_synt_N(58|110)+Pfam_Spermine_synth(113|300)+	2.5.1.16,543894,AAP97136.1,AY335900,EC 2.5.1.16,LeSPM1,NM_001247478,NM_001247478.1,NP_001234407,NP_001234407.1,Putative spermine synthase,Q7XBB3,Q7XBB3_SOLLC,SPDS3,SPM1,SPM1 protein,Solyc03g007240.2,Solyc03g007240.2.1,Spermidine synthase ,UPI00001B4E35,XM_010319081.2,XP_010317383.1,sly:543894
Solyc12g038970.1.1	4081.Solyc12g038970.1.1	Porphyromonas-type peptidyl-arginine deiminase family protein	https://smart.embl.de/smart/DDvec.cgi?smart=375:Pfam_PAD_porph(13|371)+	101267493,3.5.3.12,Agmatine deiminase,Agmatine deiminase ,EC 3.5.3.12,EMB1873,K4DEP4,K4DEP4_SOLLC,LOC101267493,Solyc12g038970.1,Solyc12g038970.1.1,UPI000276A4FC,Uncharacterized protein,XM_004252212,XM_004252212.1,XP_004252260,XP_004252260.1,XP_019067170,XP_019067170.1,sly:101267493
**adc1**	4081.Solyc10g054440.1.1	Arginine decarboxylase ; Belongs to the Orn/Lys/Arg decarboxylase class-II family. SpeA subfamily	https://smart.embl.de/smart/DDvec.cgi?smart=707:Pfam_Orn_Arg_deC_N(119|394)+	4.1.1.19,543807,A0A3Q7IH94,A0A3Q7IH94_SOLLC,ACM16841.1,ACM16842.1,ACM16843.1,ACM16844.1,ACM16899.1,ACM16900.1,ACM16901.1,ACM16902.1,ACM16903.1,ACM16904.1,ACM16905.1,ACM16906.1,ACM16911.1,ACM16912.1,ACM16925.1,ACM16926.1,ACM16967.1,ACM16968.1,AJ871577,Adc1,Arginine decarboxylase,Arginine decarboxylase ,B9V[illegible]
spdsyn	4081.Solyc05g005710.2.1	Spermidine synthase ; Belongs to the spermidine/spermine synthase family	https://smart.embl.de/smart/DDvec.cgi?smart=342:Pfam_Spermine_synt_N(53|107)+Pfam_Spermine_synth(110|298)+	2.5.1.16,544177,AJ006414,CAA07020.1,EC 2.5.1.16,NM_001247564,NM_001247564.1,NM_001247564.2,NP_001234493,NP_001234493.1,Putrescine aminopropyltransferase,Q9ZS45,SPDE_SOLLC,SPDSY,SPDSYN,Solyc05g005710.2,Solyc05g005710.2.1,Spdsyn,Spermidine synthase,Spermidine synthase ,UPI0000135DCB,sly:544177,spdsyn

nodes of NPF7.3	identifier	domain_summary	annotation	other_names
LeNRT2.3	4081.Solyc00g090860.1.1	annotation not available	https://smart.embl.de/smart/DDvec.cgi?smart=148:TRANS(67|89)+TRANS(125|147)+	543827,A0A494G9P2,A0A494G9P2_SOLLC,K4ARA7_SOLLC,LeNRT2.3,NP_001234127,Nitrate transporter NRT2.3,Solyc00g090860.1,Solyc00g090860.1.1,UPI000276198F,Uncharacterized protein,sly:543827
NR	4081.Solyc11g013810.1.1	Nitrate reductase (nad(p)h); Nitrate reductase [NADH];	https://smart.embl.de/smart/DDvec.cgi?smart=912:Pfam_Oxidored_molyb(143|322)+Pfam_Mo-co_dimer(349|483)+Cyt-b5(540|612)+Pfam_FAD_binding_6(659|766)+Pfam_NAD_binding_1(786|895)+	1.7.1.1,1.7.1.2,1.7.1.3,100736473,AEG47688.1,CAA32218.1,EC 1.7.1.1,EC 1.7.1.2,EC 1.7.1.3,F6KTI6_SOLLC,HQ616893,K4D6I5_SOLLC,NIA,NIA_SOLLC,NP_001315427,NR,Nitrate reductase (NAD(P)H) ,Nitrate reductase [NADH],P17570,Solyc11g013810.1,Solyc11g013810.1.1,UPI000276A5D9,X14060,XM_004250307,XM_004250307.1,XP_004250355,XP_004250355.1,
**Solyc01g080870.2.1**	4081.Solyc01g080870.2.1	annotation not available	https://smart.embl.de/smart/DDvec.cgi?smart=600:TRANS(46|68)+TRANS(88|107)+Pfam_PTR2(110|539)+TRANS(551|573)+	101250924,A0A3Q7EIH7,A0A3Q7EIH7_SOLLC,K4AXY0,K4AXY0_SOLLC,LOC101250924,Protein NRT1/ PTR FAMILY 7.3,Solyc01g080870.2,Solyc01g080870.2.1,UPI0002762771,Uncharacterized protein,XM_004229458,XM_004229458.1,XM_004229458.2,XP_004229506,XP_004229506.1,sly:101250924
Solyc01g090270.2.1	4081.Solyc01g090270.2.1	annotation not available	https://smart.embl.de/smart/DDvec.cgi?smart=876:Pfam_DUF630(1|59)+Pfam_DUF632(416|720)+	101257405,K4AYW8,K4AYW8_SOLLC,LOC101257405,Nitrate regulatory gene2 protein-like,Solyc01g090270.2,Solyc01g090270.2.1,UPI0002761D61,Uncharacterized protein,XP_004229746,XP_004229746.1,sly:101257405
Solyc02g021440.2.1	4081.Solyc02g021440.2.1	Cbl-interacting serine/threonine-protein kinase 23; Belongs to the protein kinase superfamily	https://smart.embl.de/smart/DDvec.cgi?smart=457:S_TKc(19|274)+Pfam_NAF(318|378)+	101259992,2.7.11.1,A0A3Q7FGM7,A0A3Q7FGM7_SOLLC,CBL-interacting protein kinase 23,CIPK23,K4B4Z4,K4B4Z4_SOLLC,LOC101259992,Non-specific serine/threonine protein kinase,Solyc02g021440.2,Solyc02g021440.2.1,UPI000276881F,XM_004231493,XM_004231493.1,XP_004231541,XP_004231541.1,XP_004231542,XP_004231542.1,sly:101259992
Solyc03g112100.2.1	4081.Solyc03g112100.2.1	High-affinity nitrate transporter 3.2; Involved in nitrate transport	https://smart.embl.de/smart/DDvec.cgi?smart=203:SIGNAL(1|24)+Pfam_NAR2(28|199)+	101245833,A0A3Q7FRX7,A0A3Q7FRX7_SOLLC,High-affinity nitrate transporter,High-affinity nitrate transporter 3.2,K4BK64,K4BK64_SOLLC,LOC101245833,Nitrate transmembrane transporters,Solyc03g112100.2,Solyc03g112100.2.1,UPI00027692F1,Uncharacterized protein,WR3,XM_004236225,XM_004236225.1,XM_010320725.2,XP_004236273,XP_004236273.1,XP_01[illegible]
Solyc06g074990.1.1	4081.Solyc06g074990.1.1	annotation not available	https://smart.embl.de/smart/DDvec.cgi?smart=526:Pfam_MFS_1(78|465)+	A0A3Q7H370,A0A3Q7H370_SOLLC,K4C9L5,K4C9L5_SOLLC,MFS domain-containing protein,Solyc06g074990.1,Solyc06g074990.1.1,UPI000276697B,Uncharacterized protein
Solyc11g013310.1.1	4081.Solyc11g013310.1.1	Auxin influx carrier; LAX3 protein	https://smart.embl.de/smart/DDvec.cgi?smart=468:Pfam_Aa_trans(40|434)+	100736478,AEI69670.1,Auxin influx carrier (AUX1 LAX family),F8SS64,F8SS64_SOLLC,HQ671065,LAX3,LAX3 protein,LOC100736478,NM_001247753,NM_001247753.1,NP_001234682,NP_001234682.1,Solyc11g013310.1,Solyc11g013310.1.1,UPI0002146BFC,sly:100736478
Solyc11g069740.1.1	4081.Solyc11g069740.1.1	annotation not available	https://smart.embl.de/smart/DDvec.cgi?smart=517:TRANS(67|89)+TRANS(338|360)+TRANS(365|387)+TRANS(394|416)+TRANS(426|448)+	Solyc11g069740.1,Solyc11g069740.1.1,UPI00027693D6
Solyc11g069760.1.1	4081.Solyc11g069760.1.1	annotation not available	https://smart.embl.de/smart/DDvec.cgi?smart=183:TRANS(5|27)+TRANS(32|54)+TRANS(61|83)+TRANS(93|115)+	101251152,A0A3Q7IZW9,A0A3Q7IZW9_SOLLC,High affinity nitrate transporter 2.4-like,K4DAD2,K4DAD2_SOLLC,LOC101251152,Solyc11g069760.1,Solyc11g069760.1.1,UPI00027693D8,Uncharacterized protein,XM_004251358,XM_004251358.1,XP_004251406,XP_004251406.1,sly:101251152

nodes of BAG5	identifier	domain_summary	annotation	other_names
HSTF24	4081.Solyc02g090820.2.1	Heat shock transcription factor, Heat shock factor 4	https://smart.embl.de/smart/DDvec.cgi?smart=301:HSF(6|99)+COIL(149|185)+	101244435,A0A3Q7FBZ5,A0A3Q7FBZ5_SOLLC,HSF4,HSF_DOMAIN domain-containing protein,HSTF24,Heat shock factor 4,Heat shock factor protein HSF24,Heat shock transcription factor, other eukaryote,K4BCN1,K4BCN1_SOLLC,LOC101244435,NM_001321450.1,NP_001308379,NP_001308379.1,Solyc02g090820.2,Solyc02g090820.2.1,UPI00027675FB,Uncharacterized
MTSHP	4081.Solyc08g078700.2.1	Belongs to the small heat shock protein (HSP20) family	https://smart.embl.de/smart/DDvec.cgi?smart=210:Pfam_HSP20(114|210)+	543507,AB017134,BAA32547.1,HSP20 family protein,LEMTSHP,MTSHP,Mitochondrial small heat shock protein,NM_001246943,NM_001246943.1,NM_001246943.2,NP_001233872,NP_001233872.1,O80432,O80432_SOLLC,Solyc08g078700.2,Solyc08g078700.2.1,UPI00000A9D03,sly:543507
Solyc01g008000.2.1	4081.Solyc01g008000.2.1	Adp-ribosylation factor 1/2; Belongs to the small GTPase superfamily	https://smart.embl.de/smart/DDvec.cgi?smart=209:ARF(1|181)+	101250243,A0A3Q7E9M8,A0A3Q7E9M8_SOLLC,ADP-ribosylation factor 1/2,ADP-ribosylation factor 2,K4ASZ8,K4ASZ8_SOLLC,LOC101250243,Solyc01g008000.2,Solyc01g008000.2.1,UPI0002762CF5,Uncharacterized protein,XP_004228551,XP_004228551.1,sly:101250243
Solyc01g066050.2.1	4081.Solyc01g066050.2.1	Tetratricopeptide repeat (TPR)-like superfamily protein	https://smart.embl.de/smart/DDvec.cgi?smart=533:TPR(411|444)+TPR(451|484)+TPR(485|518)+	101263750,A0A3Q7EFL8,A0A3Q7EFL8_SOLLC,K4AWK8,K4AWK8_SOLLC,LOC101263750,Solyc01g066050.2,Solyc01g066050.2.1,TPR_REGION domain-containing protein,UPI0002762941,Uncharacterized protein,XM_004229221,XM_004229221.1,XM_004229221.3,XP_004229269,XP_004229269.1,XP_004229270,XP_004229270.1,sly:101263750
Solyc03g115230.2.1	4081.Solyc03g115230.2.1	Atp-dependent clp protease atp-binding subunit clpb; Belongs to the ClpA/ClpB family	https://smart.embl.de/smart/DDvec.cgi?smart=911:Pfam_Clp_N(17|64)+Pfam_Clp_N(97|147)+AAA(200|345)+COIL(405|501)+AAA(598|740)+ClpB_D2-small(768|859)+	101254583,A0A3Q7FQU3,A0A3Q7FQU3_SOLLC,Chaperone protein ClpB1,K4BL25,K4BL25_SOLLC,LOC101254583,Solyc03g115230.2,Solyc03g115230.2.1,UPI0002768793,Uncharacterized protein,XM_004235918,XM_004235918.1,XM_004235918.3,XP_004235966,XP_004235966.1,sly:101254583
Solyc04g025560.2.1	4081.Solyc04g025560.2.1	annotation not available	https://smart.embl.de/smart/DDvec.cgi?smart=253:TRANS(13|35)+ARF(38|237)+	K4BR01,K4BR01_SOLLC,Solyc04g025560.2,Solyc04g025560.2.1,UPI0002765A3C,Uncharacterized protein,XM_004237204,XM_004237204.1,XP_004237252,XP_004237252.1
Solyc06g053670.1.1	4081.Solyc06g053670.1.1	annotation not available	https://smart.embl.de/smart/DDvec.cgi?smart=239:Pfam_ECH_2(13|221)+	101250512,5.3.3.8,A0A3Q7GWF4,A0A3Q7GWF4_SOLLC,Delta3-Delta2-enoyl-CoA isomerase ,EC 5.3.3.8,Enoyl-CoA delta isomerase 2,Enoyl-CoA delta isomerase 2, peroxisomal,K4C654,K4C654_SOLLC,LOC101250512,Solyc06g053670.1,Solyc06g053670.1.1,UPI0002765756,Uncharacterized protein,XM_004240966,XM_004240966.1,XM_004240966.3,XP_004241014,XP_[illegible]
**Solyc06g072430.1.1**	4081.Solyc06g072430.1.1	annotation not available	https://smart.embl.de/smart/DDvec.cgi?smart=373:IQ(70|92)+COIL(335|357)+	104648101,A0A3Q7GYQ7,A0A3Q7GYQ7_SOLLC,BAG domain-containing protein,K4C8W3,K4C8W3_SOLLC,Solyc06g072430.1,Solyc06g072430.1.1,UPI000276579A,Uncharacterized protein,Uncharacterized protein LOC104648101,XP_010322968,XP_010322968.1,sly:104648101
Solyc06g082560.1.1	4081.Solyc06g082560.1.1	Belongs to the ClpA/ClpB family	https://smart.embl.de/smart/DDvec.cgi?smart=854:Pfam_Clp_N(19|67)+Pfam_Clp_N(99|149)+AAA(199|345)+COIL(404|499)+AAA(597|759)+ClpB_D2-small(758|849)+	A0A3Q7H4E3,A0A3Q7H4E3_SOLLC,K4CAB3,K4CAB3_SOLLC,Solyc06g082560.1,Solyc06g082560.1.1,UPI00027659C2,Uncharacterized protein
Solyc07g052270.2.1	4081.Solyc07g052270.2.1	11-beta-hydroxysteroid dehydrogenase 1B-like; short-chain dehydrogenases/reductases (SDR) family	https://smart.embl.de/smart/DDvec.cgi?smart=353:TRANS(12|34)+Pfam_adh_short(52|246)+	1.1.1.-,1.1.1.146,101249837,11-beta-hydroxysteroid dehydrogenase 1B-like,11beta/17beta-hydroxysteroid dehydrogenase ,A0A3Q7I821,A0A3Q7I821_SOLLC,EC 1.1.1.-,EC 1.1.1.146,K4CFC6,K4CFC6_SOLLC,LOC101249837,Solyc07g052270.2,Solyc07g052270.2.1,UPI000276B655,Uncharacterized protein,XM_010325530.2,XP_004243840,XP_004243840.1,XP_010323832,XP_
Solyc08g066440.2.1	4081.Solyc08g066440.2.1	alpha/beta-Hydrolases superfamily protein	https://smart.embl.de/smart/DDvec.cgi?smart=404:Pfam_Abhydrolase_1(141|302)+	101250324,A0A3Q7HQX2,A0A3Q7HQX2_SOLLC,Hydrolase_4 domain-containing protein,K4CLM1,K4CLM1_SOLLC,LOC101250324,Solyc08g066440.2,Solyc08g066440.2.1,UPI000276BCA9,Uncharacterized protein,Uncharacterized protein LOC101250324,XM_004245104,XM_004245104.1,XM_004245104.3,XP_004245152,XP_004245152.1,alpha/beta-Hydrolases superfa[illegible]

nodes of DCL2b	identifier	domain_summary	annotation	other_names
AGO1-1	4081.Solyc06g072300.2.1	Eukaryotic translation initiation factor 2c; Argonaute1-1; Argonaute 1 ; Belongs to the argonaute family	https://smart.embl.de/smart/DDvec.cgi?smart=1054:Pfam_Gly-rich_Ago1(70|177)+Pfam_ArgoN(193|328)+DUF1785(337|389)+PAZ(394|526)+Pfam_ArgoL2(530|576)+Pfam_ArgoMid(586|664)+Piwi(680|1001)+	101247032,A0A3Q7HUY0,A0A3Q7HUY0_SOLLC,ACI46675.1,AFV15377.1,AFV46190.1,AGO1-1,AGO1A,Argonaute 1,Argonaute1-1,B6EBK3_SOLLC,Eukaryotic translation initiation factor 2C,FJ222764,JX467704,JX945381,K4C8V0,K4C8V0_SOLLC,LOC101247032,NM_001279128,NM_001279128.1,NP_001266057,NP_001266057.1,Protein argonaute 1A-like,Solyc06g0[illegible]
AGO2A	4081.Solyc02g069260.2.1	Eukaryotic translation initiation factor 2c; Belongs to the argonaute family	https://smart.embl.de/smart/DDvec.cgi?smart=1042:Pfam_ArgoN(215|352)+DUF1785(361|413)+PAZ(414|553)+Pfam_ArgoL2(557|603)+Piwi(710|1004)+	101249141,AFV08637.1,AFV15379.1,AGO2A,AGO2A2,Argonaute 2A,Eukaryotic translation initiation factor 2C,JX467706,JX493012,K4B7Q8,K4B7Q8_SOLLC,K4LRQ8_SOLLC,LOC101249141,NM_001279306.2,NP_001266235,NP_001266235.1,NP_001266235.2,Solyc02g069260.2,Solyc02g069260.2.1,UPI0002767EE9,sly:101249141
AGO5	4081.Solyc06g074730.2.1	Eukaryotic translation initiation factor 2c; AGO5; Argonaute family protein; Belongs to the argonaute family	https://smart.embl.de/smart/DDvec.cgi?smart=1011:Pfam_ArgoN(170|302)+DUF1785(311|364)+PAZ(369|499)+Pfam_ArgoL2(503|549)+Piwi(654|974)+	101244672,AFV15384.1,AGO5,Argonaute 5,Argonaute family protein,Eukaryotic translation initiation factor 2C,JX467712,K4C9J1,K4C9J1_SOLLC,NM_001278949,NM_001278949.1,NP_001265878,NP_001265878.1,Solyc06g074730.2,Solyc06g074730.2.1,UPI0002766790,sly:101244672
AGO6	4081.Solyc07g049500.2.1	Eukaryotic translation initiation factor 2c; AGO6; Argonaute family protein; Belongs to the argonaute family	https://smart.embl.de/smart/DDvec.cgi?smart=903:Pfam_ArgoN(48|206)+DUF1785(216|268)+PAZ(269|409)+Pfam_ArgoL2(413|459)+Piwi(556|864)+	101264839,AFV15385.1,AGO6,Argonaute 6,Argonaute family protein,Eukaryotic translation initiation factor 2C,JX467713,K4CF53,K4CF53_SOLLC,NM_001279344,NM_001279344.1,NP_001266273,NP_001266273.1,Solyc07g049500.2,Solyc07g049500.2.1,UPI000276B250,XM_010325298.2,XM_010325299.2,XP_010323600.1,XP_010323601.1,sly:101264839
AGO7	4081.Solyc01g010970.2.1	Eukaryotic translation initiation factor 2c; AGO7; Argonaute family protein; Belongs to the argonaute family	https://smart.embl.de/smart/DDvec.cgi?smart=1000:Pfam_ArgoN(148|295)+DUF1785(304|356)+PAZ(364|504)+Piwi(652|962)+	101055594,AFO53517.1,AFV15386.1,AGO7,Argonaute 7,Argonaute family protein,Eukaryotic translation initiation factor 2C,I7BBC5_SOLLC,JX047549,JX467714,K4ATU0,K4ATU0_SOLLC,NM_001279280,NM_001279280.1,NM_001279280.2,NP_001266209,NP_001266209.1,Solyc01g010970.2,Solyc01g010970.2.1,UPI0002761EDB,sly:101055594
DCL	4081.Solyc04g078850.2.1	Protein DCL, chloroplastic;	https://smart.embl.de/smart/DDvec.cgi?smart=224:Pfam_DUF3223(121|197)+	544019,AAC49433.1,AAC49434.1,DCL,DCL_SOLLC,Defective chloroplasts and leaves protein,NM_001246945,NM_001246945.1,NM_001246945.2,NP_001233874,NP_001233874.1,Protein DCL, chloroplastic,Q42463,Solyc04g078850.2,Solyc04g078850.2.1,U55219,U55278,UPI0000128FCB,sly:544019
**DCL2b**	4081.Solyc11g008520.1.1	Endoribonuclease dicer 2b; Belongs to the actin family. Belongs to the helicase family. Dicer subfamily	https://smart.embl.de/smart/DDvec.cgi?smart=1428:DEXDc(20|221)+HELICc(407|494)+Pfam_Dicer_dimer(588|673)+PAZ(844|992)+RIBOc(1013|1169)+RIBOc(1200|1353)+	101256737,3.1.26.-,A0A3Q7IQP2,A0A3Q7IQP2_SOLLC,DCL2b,EC 3.1.26.-,Endoribonuclease Dicer ,Endoribonuclease Dicer 2b,K4D5B2,K4D5B2_SOLLC,NP_001316341,Solyc11g008520.1,Solyc11g008520.1.1,UPI000276A8DF,Uncharacterized protein,sly:101256737
Solyc01g056620.2.1	4081.Solyc01g056620.2.1	Double-stranded rna-binding protein 1 isoform x1; double-stranded-RNA-binding protein 4	https://smart.embl.de/smart/DDvec.cgi?smart=288:DSRM(5|72)+DSRM(83|149)+	101261264,A0A3Q7EDA4,A0A3Q7EDA4_SOLLC,DRB4,Double-stranded RNA-binding protein 1 isoform X1,K4AVM8,K4AVM8_SOLLC,LOC101261264,Solyc01g056620.2,Solyc01g056620.2.1,UPI000276190F,Uncharacterized protein,XP_004229058,XP_004229058.1,double-stranded-RNA-binding protein 4,sly:101261264
Solyc04g049260.1.1	4081.Solyc04g049260.1.1	annotation not available	https://smart.embl.de/smart/DDvec.cgi?smart=166:Pfam_Ribosomal_S7(123|166)+	K4BRQ4,K4BRQ4_SOLLC,Ribosomal_S7 domain-containing protein,Solyc04g049260.1,Solyc04g049260.1.1,UPI0002766631,Uncharacterized protein
Solyc09g082830.2.1	4081.Solyc09g082830.2.1	Eukaryotic translation initiation factor 2c	https://smart.embl.de/smart/DDvec.cgi?smart=982:Pfam_ArgoN(135|270)+DUF1785(279|331)+PAZ(336|468)+Pfam_ArgoL2(472|518)+Pfam_ArgoMid(528|608)+Piwi(620|941)+	101259671,A0A3Q7I7U8,A0A3Q7I7U8_SOLLC,AFV15388.1,AFV15389.1,Eukaryotic translation initiation factor 2C,JX467716,JX467717,K4CVQ9,K4CVQ9_SOLLC,K4LRR5_SOLLC,K4M470_SOLLC,LOC101259671,NP_001289838,Protein argonaute 10,Solyc09g082830.2,Solyc09g082830.2.1,UPI000276C34D,Uncharacterized protein,XM_004247565,XM_004247565.1,XP_
Solyc11g021130.1.1	4081.Solyc11g021130.1.1	Belongs to the universal ribosomal protein uS7 family	https://smart.embl.de/smart/DDvec.cgi?smart=285:Pfam_Ribosomal_S7(129|279)+	K4D762,K4D762_SOLLC,Ribosomal protein S7,Solyc11g021130.1,Solyc11g021130.1.1,UPI000276966C
